# Safety of Endoscopy and Its Outcome in Pregnancy

**DOI:** 10.7759/cureus.6301

**Published:** 2019-12-05

**Authors:** Lubna Kamani, Muhammad S Achakzai, Faisal Wasim Ismail, Farhana Kayani

**Affiliations:** 1 Gastroenterology, Liaquat National Hospital & Medical College, Karachi, PAK; 2 Gastroenterology, Bolan Medical College, Quetta, PAK; 3 Gastroenterology, Aga Khan University Hospital, Karachi, PAK

**Keywords:** endoscopy, colonoscopy, pregnancy, gastroscopy

## Abstract

Objective: Gastrointestinal (GI) endoscopy is an important tool for diagnosis and treatment of GI diseases. However, when endoscopy is indicated during pregnancy, concerns about its safety for mother and fetus often arise. Our objective was to evaluate the safety and efficacy of endoscopic procedures in pregnant patients along with maternal and fetal outcomes.

Methods: This study was conducted at the Aga Khan University Hospital after Ethics review committee approval. It was a retrospective study and medical records of all pregnant patients who underwent endoscopy during pregnancy from January 2000 to January 2014 were analyzed. Data regarding the indications and type of endoscopic procedure, use of sedation and radiation were noted; data on any complications during or after pregnancy were recorded as well.

Results: A total of 48 pregnant women underwent endoscopic procedures. Procedures that were performed included gastroscopy, sigmoidoscopy, colonoscopy, and endoscopic retrograde cholangio-pancreaticography (ERCP) in 28, 15, 1, and 4 patients, respectively. The major indication for gastroscopy was hematemesis in 16 procedures (57.14%) and screening for esophageal varices was done in 10 (21.42%). The indications of ERCP were choledocholithiasis and cholangitis. However, bleeding per rectum was the main indication for sigmoidoscopy and colonoscopy. Some 34 (70.83%) procedures were diagnostic and the rest were therapeutic. Only one patient had a miscarriage in second trimester.

Conclusions: Endoscopic procedures are safe to be performed in pregnant patients in the presence of strong indications without posing major risk to the mother or the fetus. However, further prospective multicenter research studies are strongly recommended.

## Introduction

Gastrointestinal (GI) endoscopy plays an important diagnostic and therapeutic role in many clinical conditions. Patients presenting with persistent vomiting, chronic diarrhea, weight loss, iron deficiency, upper and lower GI bleed may warrant endoscopy [1-2].

Pregnancy can produce significant alterations in a human body which differ from nonpregnant state. These profound changes affect almost every system of the body including GI and hepato-biliary tract. GI symptoms can range from nausea to intractable vomiting. However, altered bowel habits, bleeding per rectum, and complicated cholelithiasis may require endoscopic treatment. When endoscopy is indicated during pregnancy, question about its effects on pregnancy outcomes often arise. Limited information is available about clinical efficacy and safety as well as maternal and fetal outcomes of such GI procedures. Endoscopic procedures are generally performed in case of strong indication preferably in the second trimester of pregnancy [3].

Data from the United States show annually more than 12,000 pregnant women have a strong indication for esophagogastroduodenoscopy (EGD), 6,000 for sigmoidoscopy or colonoscopy, and about 1,000 for therapeutic endoscopic retrograde cholangio-pancreatography (ERCP) respectively [4]. However, many potential risks are associated with these procedures during pregnancy, for example, over sedation may cause maternal hypotension, maternal hypoxia [5], arrhythmias [6], aspirations [7], and fetal hypoxia. The fetus may be exposed to potentially teratogenic drugs [8] radiation, labor, and an increased risk of premature birth.

Although safety of GI endoscopy is well established in the general population with some risks of complications [9], the potential risks associated with these procedures are not well established in case of pregnancy.

Most of the studies done for evaluating safety of GI endoscopy in pregnant patients are case series or case reports [10-11]. There is a paucity of data from Southeast Asia especially Pakistan regarding endoscopy in pregnancy. The current study was planned with the sole purpose of analyzing the safety and efficacy of endoscopic procedures in pregnant females, along with maternal and fetal outcomes, from a single tertiary care center.

The objective of the study was to evaluate the safety and efficacy of endoscopic procedures in pregnant females, and its effect on maternal and fetal outcome.

## Materials and methods

The study was conducted at the Aga Khan University Hospital Karachi from January 2000 to January 2014, after approval from Ethics review committee of the hospital. It was a retrospective study and medical records of all pregnant patients who underwent GI endoscopic procedure were retrieved from the patient management and information system (PMIS) through computer-generated codes.

The indications for procedures, medications, and radiations used during endoscopy as well as the pregnancy-related complications after the endoscopy were collected through extensive review of the nursing and doctor’s notes and endoscopy report. Pregnancy outcome and Apgar score at 1 min and 5 min were also determined from the mother delivery record.

First trimester was defined as 1-14 weeks of pregnancy, second trimester as 15-28 weeks whereas third trimester as >29 weeks of pregnancy. Term pregnancy was defined as 37 completed weeks of gestation. Infants delivered below below 2,500 g were defined as low birth weight.

A temporal relation between an adverse event and endoscopy was found plausible if the adverse event occurred within one week of procedure and was considered unlikely when the adverse event occurred after one week of endoscopy. Patients with incomplete medical record and incomplete procedure were excluded from the study. 

Data analysis

All the relevant data were entered on a proforma. Descriptive statistics were applied by using SPSS version 16. Mean and median were calculated with standard deviation (SD).

## Results

A total of 48 procedures were performed during January 2000 to January 2014. The mean age of the patients was 26.38 ± 5.5 years. Twenty-two patients (45.8%) were primigravida, the rest were multigravida. During the first, second and third trimester of pregnancy, number of procedures done were 8, 35, and 5 respectively. The mean gestational age at the time of procedure was 17 weeks (range 5-30 weeks). Out of 48 procedures, EGD, sigmoidoscopy, colonoscopy, and ERCP were done in 28, 15, 1, and 4 patients respectively. Table 1 shows major indication for EGD was hematemesis in 16 patients (57.14%) followed by dysphagia and persistent epigastric pain in three patients each (10.7%). The only indication of ERCP in four pregnant patients was choledocholithiasis and cholangitis. Bleeding per rectum was the main indication for sigmoidoscopy and colonoscopy.

**Table 1:** Indications for endoscopic procedures of all pregnant patients. EGD, esophagogastroduodenoscopy; ERCP, endoscopic retrograde cholangio-pancreatography

Endoscopic procedure	Number of patients	Number of procedures	Indications
EGD	28	16	Hematemesis
		3	Dysphagia
		3	Persistent abdominal pain
		6	Screening EGD for varices
Sigmoidoscopy	15	15	Bleeding per rectum
Colonoscopy	1	1	Bleeding per rectum
ERCP	4	4	Cholangitis and choledocholithiasis

A total of 23 procedures were done without sedation including all sigmoidoscopies, while the rest were performed under conscious sedation using midazolam in low doses 2 ± 1.5 mg. ERCP was performed under monitored anesthesia care (MAC) using propofol. Radiation was used in five procedures that is, ERCP and esophageal dilatation after proper shielding of pelvic area of the patient. The total procedure time extending from sedation till completion was less than 15 min, but the duration extended to above 15 min in procedures like ERCP and esophageal dilatation. Table 2 shows that 34 (70.83%) procedures were diagnostic and 14 (29.16%) procedures were therapeutic.

**Table 2:** Details of individualized procedures. ERCP, endoscopic retrograde cholangio-pancreatography; CBD, common bile duct

Procedures	Number of patients	Procedure performed	
Diagnostic	34		
Therapeutic	14	1	Esophageal dilatation
		4	ERCP and CBD stone removal
		4	Polypectomy
		2	Sclerotherapy for gastric ulcer
		3	Band ligation

Out of 48 patients, an immediate postprocedure-related complication was only seen in one patient (5%) with a miscarriage that occurred after esophageal dilatation in first trimester within 24 h of the procedure, whereas two patients had C-section and one had intrauterine death after eight weeks. One patient had termination of pregnancy on medical grounds because the mother was diagnosed with colon cancer after colonoscopy.

The mean weight of the babies was more than 3 kg. However, no adverse maternal events were reported following endoscopy.

## Discussion

There is limited data available on safety of endoscopy in pregnancy reported from Pakistan. Gastroscopy was the most common procedure in our study followed by sigmoidoscopy. The indications of the procedures were well defined and the American Society of Gastrointestinal Endoscopy (ASGE) guideline by Qureshi was followed [12].

Majority of the procedures were carried out under conscious sedation with midazolam, with single adverse event of a miscarriage observed within 24 h of the procedure, in a patient who underwent esophageal dilatation under fluoroscopy; no other explicable cause was found.

In our experience about 70% of the procedures were diagnostic and the rest were therapeutic. The case control study of endoscopy in the pregnancy, reported by Cappell share almost similar figures with diagnostic procedures making 79% [13], in comparison to our study, all diagnostic procedures were performed without any maternal or fetal complication.

In the current study, the outcome of the therapeutic procedures and pregnancy was good with healthy infants except one therapeutic procedure of esophageal dilatation, leading to miscarriage. Different studies have documented favorable outcomes of the therapeutic procedures and pregnancy with no complications [14]. The therapeutic procedures include esophageal sclerotherapy of varices in noncirrhotic portal hypertension and bleeding ulcers [15-19].

One of the major limitations of our study was that we had only four ERCP procedures in our study but in literature the largest series that has been reported by Tang et al. is of 68 procedures in 65 pregnant women. All the procedures were therapeutic. They reported a higher incidence of post-ERCP pancreatitis as compared to general population [20]. Similarly, in another series of 23 patients who had ERCP (therapeutic, 20 patients; diagnostic, three patients), complications included pancreatitis after ERCP (one patient), spontaneous abortion (one patient), and neonatal death at 26 h after delivery (one patient) [21]. In this study none had unfavorable outcomes. Similar results had been reported in earlier studies [22].

None of the sigmoidoscopy resulted in induction of labor or any complication. This has been supported by previous literature as cited by Cappell et al. [23] where 48 sigmoidoscopy and eight colonoscopy procedures were performed in pregnant women. The main indication was bleeding per rectum [24]. It was concluded that sigmoidoscopy can be safely performed in pregnancy and is validated to be a helpful procedure in the diagnosis of bleeding per rectum.

The other major limitation is that, it is a retrospective and single center study. Though it is a small case series and the results cannot be generalized, it is a good experience and would be helpful in planning prospective endoscopic procedures in pregnancy confidently. In future, prospective case control and randomized endoscopic studies can be carried out in this regard.

We would like to conclude that endoscopic procedures may be performed safely in pregnant patients in the presence of a strong and clear indication, without posing major risk to the mother and the baby. Potential risks, if any, should be weighed against the need for timely diagnosis and management where an underlying GI disease may be more hazardous to pregnancy than the procedure itself.

## Conclusions

Endoscopic procedures are safe to be performed in pregnant patients in the presence of strong indication without posing major risk to the mother or the fetus. However, further prospective multicenter research studies are strongly recommended.
